# General practitioners’ perceptions of the roles of community pharmacists and their willingness to collaborate with pharmacists in primary care

**DOI:** 10.1186/s40545-023-00613-5

**Published:** 2023-10-03

**Authors:** Joy Boon Ka Chong, Clivia Yao Hua Yap, Shawn Lien Ler Tan, Xuan Rong Thong, Yang Fang, Helen E. Smith

**Affiliations:** 1 Watson’s Personal Care Stores Pte Ltd, 300 Beach Road, The Concourse, #39-01/04, Singapore, 199555 Singapore; 2 Ministry of Health Holdings Pte Ltd, 1 Maritime Square, #11-25, Singapore, 099253 Singapore; 3 Lee Kong Chian School of Medicine, Nanyang Technological University, 11 Mandalay Road, Singapore, 308232 Singapore

**Keywords:** Community pharmacists, General practitioners, Interprofessional collaboration, Patient care, Primary care

## Abstract

**Background:**

Community pharmacists (CPs) have the capacity to contribute to patient care given their expertise in medication and accessibility to residents in the community. However, multidisciplinary patient care programmes where CPs collaborate with general practitioners (GPs) in patient care is rare in Singapore despite increasing healthcare demand.

**Objectives:**

This study explores GPs’ perceptions of CPs’ current roles and GPs’ ideas for and attitudes towards interprofessional collaboration.

**Methods:**

Semi-structured qualitative interviews were conducted with 20 private GPs from August to December 2020 via an online video-chat platform. GPs were recruited from the Primacy Care Research Network (pcRn), primary care networks, and using snowballing strategies. All interviews were recorded, transcribed and coded thematically.

**Results:**

Current working relationships between GPs and CPs appeared amicable but limited. GPs appreciate the existing roles of CPs: dispensing drugs not stocked in their practices and clarifying prescription details. Still, GPs appeared to rarely consider collaborative working. GPs acknowledged that CPs could enhance patient care with initiatives including medication reconciliation and advising on using medical devices. It was suggested that CPs could coordinate the purchase of drugs for primary care networks to improve GPs’ inventory management, but less enthusiasm was expressed for clinical collaborations with CPs. Major concerns about GP–CP clinical collaborations included direct competition with GPs’ own business interests, perceived low acceptability of pharmacy-led services by patients (citing extra time and cost), threat to continuity of care and the absence of a shared patient electronic health record system. Current funding mechanisms do not enable reimbursement of clinical services provided by CPs. Adoption of telemedicine technologies and governmental financial support were identified as possible enablers of GP–CP collaboration.

**Conclusions:**

GPs saw potential in CPs’ increased involvement in patient care, but perceived multiple barriers. Strategies focusing on overcoming these barriers could enable GP–CP collaboration to enhance patient care.

## Highlights

1. Multidisciplinary patient care programmes where community pharmacists collaborate with general practitioners remains a challenge in many countries despite increasing healthcare demands.
2. Inter-professionals collaborative care is hindered by direct competition with general practitioners’ own business interests; Perceived low acceptability of pharmacy-led services by patients (inconvenience and extra costs); Threat to continuity of care and absence of a shared patient electronic health record system.
3. Collaborative programmes can be encouraged by adoption of telemedicine technologies and governmental financial support.

## Introduction

Singapore is a rapidly ageing nation; one in four people is expected to be aged 65 years or more by 2030. At the same time, the old age support ratio (ratio of the working-age population (aged 20–64 years) to persons aged 65 years and over) is expected to decrease from 4 in year 2021 to 2.7 in 2030 [1]. These socio-demographic trends are going to increase the chronic disease burden and challenge healthcare. Primary care is the foundation of the healthcare system with general practitioners (GPs) and allied health professionals, such as pharmacists, playing key roles. Exploring new ways to strengthen the capacity of primary care to meet the increasing healthcare needs of the ageing population is therefore of paramount importance.

Community pharmacists (CPs) are frontline healthcare professionals who recommend and dispense medication to patients, counsel them on proper medication use, and provide drug information to the general public. In Singapore, community pharmacies and general practice clinics are predominantly independent business entities. GPs perform both drug prescribing and dispensing, while CPs dispense prescription and over-the-counter medications.

Internationally, CPs’ roles have expanded over the years from medication dispensing to more patient-centred roles such as provision of pharmaceutical care programmes. For example, in Europe and North America there are many multidisciplinary patient care programmes in the primary health care sector, including initiatives in which GPs and CPs work together to optimize delivery of patient care in the community [2–5]. Such collaborations are rare in Singapore. Instead, there is professional isolation between private GPs and CPs, even though CPs are well placed to provide patient care due to their proximity to residential areas [6]. There is no data on how often Singapore residents access their community pharmacy, but a study from North America found that on average patients visited a community pharmacy 35 times each year compared to four visits to the primary care physician [7]. Many studies have confirmed how CPs’ involvement in health promotion and in the management of common chronic diseases, (including hypertension, diabetes and hyperlipidemia and asthma) has improved patients’ medication and lifestyle adherence and health outcomes [8–12]. Such initiatives involve CPs working both independently or in collaboration with other health professionals, and patients view such extended pharmacy services as beneficial. [13–16]

In recent years, some Singapore CPs have expanded the variety of their clinical services. These include outreach medication advice and review at senior day care centres and to patients with diabetes at general practice clinics [11, 17]. Singapore CPs have also launched in-store patient care programmes to manage conditions such as diabetes and allergic rhinitis [18, 19], and provide medication reviews for patients referred from hospital-based pharmacists [14]. However, these community-based clinical services are provided in isolation with no involvement from the patient’s GPs. In contrast, hospital-based pharmacists in Singapore work closely with medical doctors within the same institution, attending ward rounds and clinics as part of the multidisciplinary team. Recently, experienced pharmacists in hospitals have been authorized to prescribe medications e without doctor signoff, thus being able to work at the top of their license [20]. These trends suggest potential for increased collaboration between private GPs and CPs in clinical service delivery.

The Singapore government recognized the need for quality healthcare services to remain accessible and affordable to a rapidly aging population [21]. One potential way of strengthening the provision of primary care is to develop greater interprofessional collaborations, and for private GPs to share their responsibilities for patient care with others. In this study, we explore GPs’ understanding and perceptions of the existing role of CPs in patient care; and the GP’s readiness, and perceived enablers towards greater GP–CP collaborations.

## Methodology

### Study design, setting and population

One-to-one semi-structured qualitative interviews were conducted with 20 practising private GPs in Singapore. This number is appropriate for qualitative studies where the purpose of the research is to collect rich data and obtain insights into the participants’ perceptions [22]. The study was open to practising private GPs in Singapore.

### Recruitment

Participants were recruited from the Primary Care Research Network (pcRn) hosted by Lee Kong Chian School of Medicine, Nanyang Technological University Singapore. The network was set up to promote primary care research in Singapore and membership is open to all GPs in Singapore. Snowballing strategies were also employed because GPs are a professional group where direct access is limited. Interested participants were sent the study information sheet and were able to have their questions addressed. Informed consent was obtained before the interviews were conducted and all participants received a token of appreciation.

### Data collection

All interviews were conducted over Zoom in English by two medical students who were trained in qualitative interviewing techniques. Each interview began with a series of brief questions, including the participants’ professional experience and basic characteristics of their practice. Following that, questions listed in Table 1 were asked to understand better their perceptions of CPs, the possibility of GPs collaborating with CPs, their perceptions of CPs expanding the scope of their clinical involvement and factors that facilitate or impede GP–CP collaboration. Questions asked were broad and open-ended in nature to avoid restricting the scope of the answers provided. [23]

**Table 1:** Question guide and probe

1	Can you describe your experiences with community pharmacists in primary healthcare?
2	How do you think the roles of community pharmacies and pharmacists have changed over time?
3	What specific roles for the community pharmacists in primary health care would you like to see developed?
4	What are your views regarding collaborative clinical service between GPs and community pharmacists?
5	Are there any patient care areas in which you and your practice would like to collaborate with community pharmacists?
6	In some countries, community pharmacists play a key role in delivering health care in the community; they lead clinical services and are involved in providing many interdisciplinary clinical services. In your opinion, could similar initiatives be introduced in Singapore to improve patient health management?
7	What do you see as the major advantages and disadvantages of clinical services led by community pharmacists?
8	Are your views different for interdisciplinary care programmes that involve community pharmacists?
9	What factors are important for developing successful collaborations between community pharmacists and GPs?
10	What might be the barriers to developing such collaborations? And how might these be overcome?

All interviews were audio-recorded digitally and transcribed before analysis.

### Data analysis

The transcripts were analysed using thematic analysis as the theoretical framework [24]. Given the limited literature on our research questions, this method allows us an understanding of the nature of GPs’ perceptions of CPs  with less bias related to preconceived notions [25]. Each transcript was analysed by at least two authors independently [26]. The transcripts were first read to gain a general idea [27], and then read carefully, and repeatedly, to identify words and phrases expressing relevant ideas and perceptions; these themes became codes for this analysis [28]. The relationship between codes was examined, enabling the creation of themes and subthemes [29]. Theme saturation was also observed in his study. In the results verbatim quotes are used to illustrate the themes [30].

### Ethics approval

Ethics approval was obtained from the Nanyang Technological University Institutional Review Board (IRB-2019-11-019) before the commencement of the study.

## Results

The GPs interviewed practised in different parts of Singapore, some in predominantly residential areas and others in the city centre, serving patients with varied demographic profiles. Each interview lasted approximately 30 min. There gender split was fairly even; slightly less than half the participants had more than 20 years of practice experience (Table 2). Five major themes and 19 subthemes were identified from the interviews (Table 3). The major themes were (1) GPs’ perceptions of the role of CPs in primary care; (2) barriers to GP–CP collaboration; (3) perceived resistance of patients to an increased role of CPs in their health care; (4) changes needed in the organization and funding of the healthcare system to facilitate greater GP–CP collaboration; and (5) potential areas for future GP–CP collaboration.

**Table 2:** Demographic and practice information of the general practitioners (n = 20)

Demographic/practice information	Number of general practitioners (%)
Gender	
Male	11 (55%)
Female	9 (45%)
Practice experience (years)	
< 5	0
5–10	6 (30%)
11–15	2 (10%)
16–20	3 (15%)
> 20	9 (45%)

**Table 3:** Summary of themes and sub-themes

**GPs’ perceptions of the role of CPs in primary health care**
Traditional role in dispensing prescribed medication
Awareness of other CP roles
Limited GP-CP interactions
**Barriers to GP-CP collaboration**
Little recognition of benefits of collaboration
Uncertainty about CPs’ competence in clinical services
Poor potential for professional relationship continuity
GPs’ reluctance to share their professional role
CP role expansion seen as a threat to GP clinic income
**Perceived resistance of patients to an increased role of CPs in their health care**
Inaccessibility
Reluctance to pay more
**Changes needed in the organization and funding of the healthcare system to facilitate greater GP–CP collaboration**
Facilitation of economies of scale within general practice
Greater investment and changes in funding
A shared electronic clinical record
**Potential areas of GP practice in which CPs can contribute**
Coordinating the purchase of medications
CPs as continuing medical educators
CP-staffed helpline for GPs
Medication reconciliation
Chronic disease management
Health promotion and screening

### GPs’ perceptions of the role of community pharmacists in primary health care

#### Traditional dispensing of prescribed medications

In all the interviews GPs described the role played by CPs in dispensing prescribed medications to patients—medications that were not included or stocked in the general practice’s own formulary. In this context some GPs also referred to how the CP might need to contact them to clarify details on the prescription, for example dose or frequency, or to ask about the acceptability of a substitute medication.

“For the prescription, the patient will bring the prescription to the [pharmacy chain] and then they prescribe the medication that our clinic doesn't carry. If they don’t have this medication in their pharmacies, they will call up and say... can we substitute that?” (GP 1, M).

Some GPs described how CPs also are a source of advice for their patients on over-the-counter medications and supplements. The longer opening hours of community pharmacists in comparison to their own clinics enhanced the importance of this role.

“Their [community pharmacies’] opening hours are really quite long, so they provide a need for easy accessibility to common cold medicines and maybe skin treatment.” (GP 7, F).

CPs were also valued as a source of instruction on the use of medical devices and equipment, for example:

“Sometimes I want to send a patient to buy asthmatic medicine, like an inhaler, a child would need a spacer. I would need the pharmacy to teach. So, I put … on a prescription, spacer times one, please teach patient how to use.” (GP 5, F).

#### Awareness of other CP roles

Many GPs also spoke of their awareness of CPs involvement in roles other than traditional dispensing, such as providing information on drug availability and conducting medication management reviews for nursing homes.

“I just call him [the pharmacist], and say, this patient from Croatia has this medicine in front of them. Do you think this is in the Singapore market? Then they help me check.” (GP 5, F).

“They are mainly doing things like nursing home medicine audits… you could say that’s non-traditional dispensing work.” (GP 15, M).

Fewer GPs spoke about CPs involvement in lifestyle or health related counselling, for example in the context of smoking cessation, chronic disease management and lifestyle.

“But probably other … sort of work they are being asked is things like smoking cessation, some healthy living sort of counselling, medication counselling sometimes” (GP15, M).

#### Limited general practitioner–community pharmacist interactions

In general, the GPs perceived CPs as helpful and pleasant. However, many of the GPs reported having no or little interaction with CPs.

“Actually, working with them is very minimal contact, because what we do is when I don’t stock a particular drug in my clinic, then I'll write a prescription and I'll pass it to the patient who goes to a community pharmacist.” (GP 6, M).

One GP contrasted this amount of interaction with a pharmacist with his experience when working in a hospital setting, where there were plentiful interactions and pharmacists were easily contactable for drug information. This GP expressed a desire for more interaction between GPs and pharmacists in the community setting.

“So, we don’t really have a lot of interaction… when I was in the hospital, I felt pharmacologists (sic) are outstandingly amazing. I thought they are absolutely wonderful. Uh, call them at 3am to say, what antibiotics do I use for someone allergic to penicillin who has pseudomonas.” (GP 14, M).

### Barriers to general practitioner—community pharmacist collaboration

Prior to the interview GPs appeared to have given little thought to ways of increasing collaboration, and some were uncertain not only how, but whether fostering such collaborative relationships were even necessary.

#### Little recognition of benefits of collaboration

GPs in general did not perceive pressing need for collaboration with CPs or support from them in patient care, with statements such as “I’m not sure how to increase the collaboration and I’m not sure if there is a need to.” (GP 10, F) Whilst recognizing that CPs may be able to provide some counselling and advice on disease management, their preference was for this to be provided by a primary care nurse, and that they themselves can manage any drug-related queries using their clinical experience, online searches and continuing medical education.

“I really don't see an additional role, at this point in time, to get them [community pharmacists] to come in. Ah, of course, we have, like health services we do make use of, for example, nurse counsellors for smoking cessation, for asthma counselling, for diabetic retinopathy screening and diabetic foot screening. So, these are all outsourced already.” (GP 9, M).

#### Uncertainty about CPs’ competence

Although most GPs appreciated CPs superior knowledge about medications and prescribing, two GPs were unsure about their clinical capabilities with patients. One GP recalled his medical training and expressed concern that CPs are not prepared for patient interactions and counseling.

“I think those days, like way back, when I was in medical school... pharmacists are just taught to dispense... and the doctor will take care of everything else. So, I’m not sure about the younger pharmacists who are out there now, whether they are ready to, take on the, the challenge of attending to patients.” (GP 3, F).

“For lifestyle changes, I would go for a diabetic nurse instead of a pharmacist … I think the diabetic nurse is specialized in this area of diabetic counselling, so it’s very targeted for patients. But the pharmacists may not be so detailed. What the pharmacist can counsel patient on might be what I can also counsel the patient on.” (GP 2, F).

#### Poor potential for relationship continuity

A GP described the difficulty building a long-term relationship with any CP because they often move between pharmacy chain outlets.

“I am in [location] … where just downstairs is the MRT [Mass Rapid Transit] station. So, I can just walk down to MRT and say hello to the pharmacist … I've been here so many years, I knew a few of them, but unfortunately, they keep on moving them around, so I don’t have a long-term relationship with any of them.” (GP 6, M).

#### GPs’ reluctance to share their professional role

Several GPs implied reluctance to relinquish any control of their patients’ care to pharmacists despite valuing pharmacist’s knowledge and expertise. GPs also argued that they need to remain in control of patient’s care as they feared they would be the one’s bearing the medical liability in any GP–CP partnership.

“The pride of being a family physician is that we provide comprehensive mix of services right to the patient. That means we take care of the holistic aspect. So, if you are going to be sending the patient away to a community pharmacy or pharmacies, right, to look after him or her, then I’m just wondering... if that might affect the comprehensiveness of care providing.” (GP 7, F).

“So, there's a lot of other issue that can occur in the complex patient kind of thing. So, who is gonna take charge of all these things? Are the pharmacists going to say that, okay, I stop this medicine, uh, if the patient gets a stroke, it's my fault. Now what is the liability or integrity?” (GP 15, M).

#### CP role expansion seen as a threat to GP clinic income

Another barrier to collaboration was related to the financial viability. GPs highlighted that private GP clinics, especially those situated in residential areas, already face considerable pressures to lower their consultation fees, and the dispensing of medication enables them to remain financially viable. Reducing their medication dispensing and sharing chronic disease patient care may reduce their income.

“I have worked in heartland [local term to describe residential areas with public housing where > 80% people live] General Practices before and I don’t think that it’s [GP–CP collaboration] going to happen, to be honest.... Which Ah Ma [older woman] is going to pay, like $20 consult, like, you know, plus another $10 for a prescription fee, yes … There’s a lot of downward pressure on the consultation fee, right. A lot of GPs, in heartlands, they are charging, like, what? $8, $10 consult … So, what do you do? You sort of stock all the medications that those patients generally want. Then you mark it up, right? And then they sort of, go through with subsidizing your loss in consultation fees … So, … the problem of your collaboration thing … it’s not going to happen. If it is going to happen, then half of the GP clinics in, in HDB town will close, because they can’t break even anymore.” (GP 5, F).

It was recognized that sustainable collaboration requires there to be mutual benefit to both GPs and CPs, one GP explaining:

“So, it has to be something that, uh, benefits the GP in terms of, like, what’s in it for him, right? And also, what’s in it for the pharmacist … I think that if you want to work with a private GP sector, right, I think you have to be very conscious that they don't see you as cutting into their pie.” (GP 7, F).

### Perceived resistance of patients to an increased role of CPs in their health care

GPs perceived that their patients have greater trust in their doctors than pharmacists, as illustrated in the following quote.

“They may not feel as comfortable with the pharmacist giving information versus, let’s say, coming from a doctor themselves.” (GP 3, F).

#### Inaccessibility

Most GPs thought that patients, especially those with limited mobility, would not be keen on CP-led services because of the inconvenience of attending for healthcare in more than one location. GPs thought that the familiarity and convenience of the GP clinic as a ‘one stop shop’ would reduce patients’ willingness to make an additional visit to a pharmacist for medication-related advice or lifestyle counselling.

“Aunty, uncle is not going to walk, another 300 m down on the aching knee. To go and talk to the pharmacist just… Just so it’s collaboration, you know. They, they would prefer to do everything under one stop.” (GP 5, F).

Some GPs suggested that the use of telecommunication to deliver CP-led services may be a possible way forward and would minimize the physical access barriers for patients. However, some GPs were more reticent, expressing concern that such initiatives could only benefit younger patients, excluding older patients and others less tech savvy.

“I think if patients are willing to get counselling through telemedicine and tele-counselling through the pharmacist, I think that is an option we can explore. If we say, for example, I have no time to counsel the patient. I can always give the patient … a web link … so that they can contact the pharmacist and do, like, a Zoom meeting, like what we’re doing now. I think that’s something that can be explored, but no one has thought about it yet … I believe this is already ongoing in the polyclinics, okay, tele-counselling by allied health, by in-house pharmacists is already going on. But it's not done in the private setting, yeah.” (GP 9, M).

“Okay, telemedicine is useful for the younger ones. The older ones? … we have this PRPP [a web-based portal] and I had like one person log in three times. Or the old folks can’t even log in, they can’t even handle the phone, my staff have to do it for them.” (GP 17, F).

#### Reluctance to pay more

Many GPs did not think that patients would be willing to bear the additional cost of additional CP-led healthcare services, which are not covered by their health insurance policies.

“Okay, of course I mean for any kind of services for the patients they are probably most concerned about costs, right. So, if they have to pay a certain amount for this [CP-led] extra service then they may not be so willing to.” (GP 4, M).

“I mean, [hospital] outpatients [pharmacy] are different, right…you just send that patient to the smoking cessation clinic and make them listen, whereas for us it’s a bit harder... to tell patients to do this and that. And of course, again, whether there’s a cost. I mean if it’s free, maybe they would. But if you have to pay some additional cost to do that, yeah, that will also be the other issue.” (GP 19, F).

### Changes needed in the organization and funding of the healthcare system to facilitate greater GP–CP collaboration

#### Facilitation of economies of scale within general practice

The competing business interest between GPs and CPs was considered an almost insurmountable challenge for collaboration; a referral made to a community pharmacist may potentially lead to a loss of income and the patient.

“You would consider that you are, actually sort of, referring your patient away to another business entity. So, they would take away your patients.” (GP 8, M).

The development of a partnership between a small or solo GP clinic and a community pharmacy was not considered practical because of the clinic’s limited scale of operation.

“For small practices like mine, which applies to most of the GP clinics in Singapore, the operating costs will be fairly low, so we don’t usually engage pharmacists to work in the clinic. Usually, the pharmacist will be working for hospitals, polyclinics where they see large groups of patients and they need to handle large amounts of medication. So, there will be like an economy of scale where the use of pharmacist will be more in those kinds of settings. Whereas in a clinic setting we will need someone who’s able to do a bit of everything, … So, they are bit like a nurse and a pharmacist, all in one, that kind of role… Yeah, so, to make it affordable for patients” (GP 4, M).

Some GPs spoke about the potential to achieve economies of scale through a network of clinics. The linking of private general practices through an umbrella organization, such as a primary care network, was seen as a possible way of achieving access to a CP and the expertise that a clinical assistant was unable to provide, for example the optimization of drug therapy.

“You can tie up the CP to the primary care network. That might be helpful in terms of increasing the comprehensiveness of the services, but you don't necessarily fragment the care. A way to do is to using existing networks to link up CPs and GPs.” (GP 7, F).

#### Greater investment and changes in funding

Respondents recognized the need for government backing and additional funding as perhaps the most important factor needed to facilitate adoption of any CP-led services for primary care patients. Coverage of expenditure would be needed for patients using both public and private health care.

“The problem is because like for my current place [of practice], they use a lot of corporate insurances. They have a lot of company issued insurance card. So, such pharmacy services, usually it’s not claimable... if they actually start to include pharmacy services as one of the possible things that can be claimed, then I think that will definitely help a lot… they don’t have to feel that they are forking out extra cash for the extra services.” (GP 3, F).

#### A shared electronic clinical record

GPs spoke about how CPs have access to only partial information about the patient’s medical problems, limiting their ability to provide holistic care or to rationalize medications. A shared electronic record is needed for the exchange of information and alerts the other party to changes in medication. To create this system, clinics using paper records would need to adopt electronic health records, and the issues of data access and security need to be addressed.

“They [the pharmacists] are not going to talk about the high blood pressure of the patient, what to drink and all these things. They may not know. Basically, the patient just goes there and buys the medicine. And the pharmacy will just advise the patient on that medicine alone. Because she's not prescribing other medicine, right? She may not know what the patient's taking as well.” (GP 15, M).

“So, like if you want continuity of care, right, there must be a way to share the information,... you have to think about how to transfer information. So, your transfer of information cannot be paper, right? You have to move from paper records.” (GP 7, F).

“So, if I update the medications, okay, the GPs are informed. And then there must be a way to alert the GP. I mean, the GP is not going to be always checking the NEHR^1^ to see whether the medicine has been updated or not, right?... Then you’re going to have another app, it cannot be that there are so many apps now. How will you manage?... Okay, so then you have your GP clinics who are not converted to electric medical records... so you have to think about that as well… you have to think about security.” (GP 7, F).

### Potential areas of GP practice in which CPs can contribute

#### Coordinating the purchase of medications

Despite the obstacles highlighted above, opportunities for GP–CP collaboration to improve patient care were suggested by the GPs. An example very commonly mentioned was for CPs to coordinate the purchase of medications rarely prescribed within each clinic and may expire before being dispensed.

“Because our clinic we are seeing wastages also because you buy in bulk then you don't use, no patient wants, then you just throw away after expiry. So, if we don’t have any of this kind of medication, then we just ask the patient to go over to the pharmacy. I think if they can support more clinics, they can buy more in bulk.” (GP 16, F).

#### CPs as continuing medical educators

Whilst it was not viable for a clinic to have a part-time of sessional CP working in their clinic, other ways for CPs to share their knowledge were proposed. Some GPs expressed interest in CP-led continuing medical education seminars (CME) about drug interactions and medication guidelines, the latter topic was considered as particularly challenging as these are frequently updated.

“Why don’t they promote their image and give us some CME seminars. For example, as doctors, we are very interested in drug interactions and area that we may not be so good at. For example, pharmacists can give us talks on psychosis, depression medications, latest drug interactions. We will also be interested in drugs that [are prescribed] from hospitals.” (GP 2, F).

#### CP-staffed helpline for GPs

Often the GPs suggestions for new initiatives can ‘raise CPs’ profile’ in addition to being service development. The introduction of a helpline for GPs to access advice on complex drug-related matters can perform these functions in addition to improving patient care.

“Um, but in the complex cases, there are some medications, some patients are [on] polypharmacy, and it's quite complicated. Like you know, Madopar and lots of medication, whereby we think that a pharmacist input will be useful in terms of dose adjustment, like having a, it’s like a family pharmacist, right? … When you don't know how to, prescribe a drug, they don’t have such a service… this will be helpful? Yeah, like a helpline, I guess.” (GP 18, M).

How such a service could be funded was unclear, there was an apparent reluctance of GPs to invest in this from their clinic’s income.

“If there is a cost, I won’t use it … If you [have to] pay $10, I won’t call.” (GP 12, M).

“As long as you don't mandate that, you know, I have to pay a subscription of $50 a month every, every month for this helpline number to call if you need.” (GP 18, M).

#### Medication reconciliation

Engaging CPs in medication reconciliation was another service for development with CPs and provable clinics, building on the CPs expertise in pharmacology to optimize prescribing and to minimize medication-related errors, particularly in the elderly with multiple chronic disorders.

“You know, a lot of times our chronic care patients has lots of medicine. And it becomes a mess after each [hospital] admission. There will be more and more, there are a lot of changes in the medicine sometimes. Sometimes the doctor just changes everything around so, it's very, very confusing and … [they] give a patient a big packet of medicine, [and] they go home. Then they have the packet from the last consult from another doctor. It becomes very, very difficult for the patients or their family to reconcile this, all this medicine stores … So, it's good that pharmacists … maybe go into the homes to look at, some of this medication, and perform some medication reconciliation …” (GP 15, M).

#### Chronic disease management

Many GPs were unfamiliar with the local CP-led clinical services, such as diabetes and allergic rhinitis management. When probed for their views, GPs saw potential utility in chronic disease management, since CP may be able to spend longer time with a patient providing training on the correct use of medical devices.

“Because most of our diabetic education in Singapore is either in restructured hospitals or the polyclinics… so, in terms of private practice, … there’s a place for the community pharmacist.” (GP 19, F).

“If there is something where I can give a prescription to get patient to go to pharmacy and …. the pharmacist can teach them about all the asthma things that they should do, how to use the inhaler properly, how to use everything properly, that would save me time because often, I actually have to teach the patient the various inhaler devices they have and get them to know how to do it properly in the clinic … I think for certain, certain chronic conditions which require a lot more work... if they’re able to help, there would be a much greater benefit. If the pharmacy actually can take over that role and, I’m more than happy to pass on this role to them.” (GP 13, M).

#### Health promotion and screening

CPs’ potential role in health promotion and disease prevention was mentioned far less, and when mentioned it was more in the context of “simple” screening programmes (e.g., hypertension and diabetes) and promoting vaccination programmes, rather than undertaking vaccination activities.

“Yeah, they can do periodic screening for people for their height and weight, you know, simple stuff, you know? Then do periodic screening for blood pressure, like blood glucose, simple stuff.” (GP 12, M).

“… because of their unique positions and catchment areas, they can help to educate in terms of vaccinations, you know? Like when they are dispensing medicines and all that, right? … They see a large group of people and, they can say, oh you know, have you done your ‘flu jab? Why don't you see a doctor for your flu jab?” (GP 7, F).

## Discussion

This study described GPs’ perceptions of CPs’ current and potential role in patient care, elucidated GPs’ perceived barriers to working with CPs, and obtained insights on overcoming these barriers to promote GP–CP collaborative practice. Current working relationships between GPs and CPs appeared to be amicable but limited. GPs generally had a positive opinion of CPs and were appreciative of their role dispensing drugs not stocked in their own clinics, answering their drug-related queries, and clarifying prescription details. However, GPs had minimal awareness of CPs’ potential role in providing clinical services beyond dispensing medications and advising on over-the-counter medications.

Amongst the GPs interviewed, the idea of collaborating in patient care with CPs did not appear to be high on their agenda. Even if they could appreciate the potential benefits of GP–CP collaborative care, they moved quickly to discussing the impracticalities, including the increased costs of engaging CPs’ expertise in patient care and weighing these against the benefits. GPs saw the increase in clinic operating cost of CP service-integration would ultimately need to be borne by the patient, and consequently they anticipated a low patient demand for CP services, a demand which would only be modified by the reimbursement of CP services by health insurance policies or financial subsidies from the government. This concurs with the views on CP service funding raised by GPs as a challenge in studies from Australia, Canada and New Zealand [31–33]. Government commitment, including financial support, are necessary for enabling the development of GP–CP collaborations that are cost-neutral to all parties [34].

In addition, GPs anticipated patients would be resistant to CP consultation because of the inconvenience (time and distance) of any non-GP-based clinical service. To minimize this GPs suggested a CP could be peripatetic, moving around a network of private clinics on a sessional basis. Telemedicine technologies, such as video conferencing for tele-counselling sessions, were also proposed as a way of reducing the inconvenience of CP-provided care for patients.

Many countries are already moving forward with expansion of pharmacists into primary care teams, and a recent systematic review of 36 studies done predominantly in Europe, North America, and Australasia found that barriers to collaboration included GPs perceiving CPs as lacking knowledge and skills regarding patient care [35]. Other barriers included GPs’ concerns about CPs’ training and clinical competency. These perceptions overlapped with the reservations reported in our study. Our interviewees’ perceptions of CPs’ skill and expertise varied, and GPs with positive perceptions were open to CPs engagement in medication reconciliation, patient education in the use of devices, chronic disease management and health promotion.

Other barriers to collaboration included GPs’ hesitancy to involve CPs in delivering patient care due to medicolegal concerns. This concurs with Gordon et al.’s finding on GPs’ concerns about losing control in disease management of their patients [36]. GPs’ preference to counsel patients on their own instead of delegating the task to CPs has also been echoed by another study in New Zealand, which reported GPs’ fear about patient confusion when involving pharmacist in medication review [37].

GPs in our study were concerned that CPs’ limited access to patient medical information would constrain their ability to add value to patient care. This concurs with a Canadian finding where GP cited lack of electronic medical record interoperability as one of the barriers integrating a co-located clinical pharmacist in a medical clinic [38]. In Singapore, National Electronic Health Record (NEHR) was introduced in 2011 to collect summary of patient health records across different healthcare institutions, enabling authorized healthcare professionals to upload and view patient’s health data. However, less than a third of private healthcare providers, including GPs, utilize NEHR [39]. Increasing the enrolment into the NEHR amongst GPs in Singapore and making these easily accessible to CPs will enhance CPs’ ability to provide clinical services.

This study highlighted some areas in which GPs warmed to future GP–CP collaboration. These areas include drug information service for doctors and coordination of drug supplies to GP clinics. These preferences are not dissimilar to preferences expressed in other countries. Kelly et al.’ work with Canadian GPs, which found a preference for further collaborations with CPs on the facilitating medication insurance claims and promoting medication adherence, more than to receive advice on patient drug therapy (e.g., medication selection, dosage adjustment) [40]. A study in New Zealand found GPs to show a stronger acceptance of CPs’ non-clinical roles than clinical roles [41].

To facilitate future GP–CP collaborations requires enhancing GPs’ knowledge and perceptions of CPs’ capacity to deliver clinical services beyond their traditional roles. This is a priority for professional bodies and individual CPs. In the longer term, clinical services in the community could be supported by greater emphasis in the professional education of doctors and pharmacists on multidisciplinary collaboration and the design of innovation in practice [42–44]. Implementation of the “Healthier SG” initiative in Singapore is an opportunity for fostering collaboration between GPs and allied healthcare professionals, including pharmacists, enhance the quality of patient care by leveraging the expertise and contributions of different healthcare professionals [45]. Government commitment, including financial support, is also necessary for aligning financial incentives and developing GP–CP collaborations that are cost-neutral to all parties. Furthermore, increasing the enrolment into the NEHR amongst GPs in Singapore and making these easily accessible to CPs will enhance CPs’ ability to provide clinical services.

To our knowledge, this is the first study in Singapore investigating GPs’ perceptions of CPs. A key strength of this study is that the interviews were conducted by medical students rather than pharmacists, which encouraged GPs to share their views freely. The methodology used was rigorous, all audio transcripts were coded and analysed thematically by a minimum of two researchers independently. However, only GPs were recruited to participate in this study, and no other stakeholders were interviewed. Doctors talked about what they perceived as the barriers for their patients, and whilst this information was second hand it was very similar to what consumers in a Malaysia study expressed [46]. Future research in Singapore is needed to explore CP–GP collaborations with patients,  CPs, senior managers and policymakers. The creation of this holistic view will facilitate a better sense of how best to move forward. Moreover, detailed participant demographics were not collected for analysis. We acknowledge that this limitation may impact the generalizability or transferability of our findings. However, the themes generated in this study may be further investigated in a larger study to assess if they are generalizable to the wider population of Singapore private GPs.

## Conclusion

In conclusion, GPs saw potential benefits in CPs’ increased involvement in patient care but they were lukewarm towards collaborating with CPs in their clinical roles. Barriers pertaining to the implementation of CP–GP collaboration were perceived low demand from patients for CP services due to additional cost, time, inconvenience, GP concerns about relinquishing control, and constraints in the current healthcare system (funding model, electronic health records). Interventions and strategies focusing on these factors, notably government funding or subsidy, could enable interprofessional collaborations between primary care physicians and CPs to enhance patient care.

## Data Availability

The datasets generated during and/or analysed during the current study are not publicly available as consent to release audio transcript was not obtained participants but are available from the corresponding author on reasonable request.

## Footnotes

1 National Electronic Health Record, the local system of electronic medical records that is shared between participating healthcare institutions.
